# Cupricyclins, Novel Redox-Active Metallopeptides Based on Conotoxins Scaffold

**DOI:** 10.1371/journal.pone.0030739

**Published:** 2012-02-03

**Authors:** Marco Barba, Anatoli P. Sobolev, Veranika Zobnina, Maria Carmela Bonaccorsi di Patti, Laura Cervoni, Maria Carolina Spiezia, M. Eugenia Schininà, Donatella Pietraforte, Luisa Mannina, Giovanni Musci, Fabio Polticelli

**Affiliations:** 1 Department of Biology, University Roma Tre, Rome, Italy; 2 Institute of Chemical Methodologies, CNR, Monterotondo Stazione, Italy; 3 Department of Biochemical Sciences, Sapienza University of Rome, Rome, Italy; 4 Department of Cell Biology and Neurosciences, Istituto Superiore di Sanità, Rome, Italy; 5 Department of Drug Sciences and Technologies, Sapienza University of Rome, Rome, Italy; 6 STAAM Department, University of Molise, Campobasso, Italy; 7 National Institute of Nuclear Physics, Roma Tre Section, Rome, Italy; University of Crete, Greece

## Abstract

Highly stable natural scaffolds which tolerate multiple amino acid substitutions represent the ideal starting point for the application of rational redesign strategies to develop new catalysts of potential biomedical and biotechnological interest. The knottins family of disulphide-constrained peptides display the desired characteristics, being highly stable and characterized by hypervariability of the inter-cysteine loops. The potential of knottins as scaffolds for the design of novel copper-based biocatalysts has been tested by engineering a metal binding site on two different variants of an ω-conotoxin, a neurotoxic peptide belonging to the knottins family. The binding site has been designed by computational modelling and the redesigned peptides have been synthesized and characterized by optical, fluorescence, electron spin resonance and nuclear magnetic resonance spectroscopy. The novel peptides, named Cupricyclin-1 and -2, bind one Cu^2+^ ion per molecule with nanomolar affinity. Cupricyclins display redox activity and catalyze the dismutation of superoxide anions with an activity comparable to that of non-peptidic superoxide dismutase mimics. We thus propose knottins as a novel scaffold for the design of catalytically-active mini metalloproteins.

## Introduction

The rational redesign of (macro)molecules based on stable scaffolds tolerant to multiple amino acid substitutions is a powerful tool to develop novel catalysts with potential biomedical and biotechnological applications [1]–[6]. A class of (macro)molecules which display these characteristics is the knottins family of disulphide-constrained peptides [4]. These miniproteins, tipically around 30 amino acids in length, share a peculiar knotted topology of three disulfide bridges [7]. This topology is observed in a number of evolutionary and functionally unrelated protein families including plant protease inhibitors, cone snails, snakes and spider toxins, and EGF-like domains [8]. The main structural features of knottins are their stability to thermal denaturation, resistance to proteolytic digestion, due to the cystine knot, their small size, which makes feasible their production by chemical synthesis, and a high tolerance to sequence variation of the intercysteine loops [4]. Knottins thus represent optimal scaffolds for redesign strategies aimed at developing novel metal-based catalysts.

Among knottins, the highest tolerance to sequence variability is observed in conotoxins, neurotoxic peptides from cone snails venom, frequently cited as an example of natural combinatorial chemistry [9].

In a previous study, we tested the potential of a natural 9 amino acids-long, disulphide-constrained peptide, named Contryphan-Vn [10]–[12], as a scaffold for the development of redox-active mini metalloproteins by computational design of two variants carrying a four-His copper binding site. The redesigned peptides, named Cupryphans, were synthesized and characterized by a variety of spectroscopic techniques, demonstrating that they selectively bind Cu^2+^ with a fairly high affinity and are endowed with superoxide dismutase activity [5].

The present study extends the work carried out on Contryphan-Vn to longer peptides, with the goal of finely modulating the stability and the catalytic activity of the redesigned metalloproteins by having a higher number of amino acid positions available to substitution. In particular, we exploited the high tolerance of conotoxins to multiple amino acid substitutions of to engineer a copper binding site in two variants of an ω-conotoxin. The design procedure took into account the position-specific conservation of the intercysteine loop residues in this family of peptides in order to facilitate spontaneous achievement of the right fold. The rationale behind this choice was that of keeping the composition of the intercysteines loop as close as possible to that of the natural sequences to favour loops conformations close to those observed in ω-conotoxins. Furthermore, the peculiarity of this work with respect to other redesign strategies employing the knottins scaffold is that the four His metal ligands were introduced in the place of four of the six Cys residues of the natural scaffold, with the aim of avoiding multiple conformational isomers arising from incorrect cysteine pairing during oxidative folding of the peptides.

## Results and Discussion

### Rational design of a copper binding miniprotein based on conotoxins scaffold

**Figure 1:**
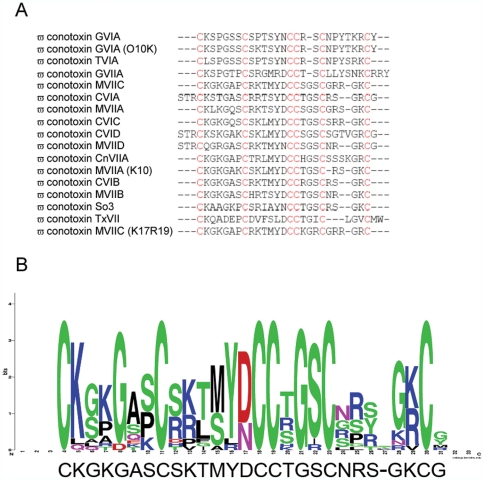
Amino acid sequence alignment of ω-conotoxins (A) and Weblogo [**17**] plot (B). The consensus sequence of the aligned ω-conotoxins is reported below the plot in panel B. The consensus sequence has been obtained by calculating the frequency of each amino acid in each position of the amino acid sequence of the aligned ω-conotoxin sequences using the Weblogo tool [17].

The strategy employed in the present work to select a suitable scaffold for engineering a mini metalloprotein was that of deriving a consensus sequence from one of the most studied knottins families, the O superfamily of conotoxins [13]. The rationale was that of identyfing a scaffold with sequence/structural properties common to all the members of the family and thus able to spontaneously attain a stable fold. The amino acid sequence of one of the members of this family, the ω-conotoxin GVIA, which is one of the best characterized in terms of both a function and structure [14], was used as a bait in a *PSI-BLAST* [15] search of the non redundant protein sequences database to recover all protein sequences displaying a significant sequence similarity. The recovered amino acid sequences were then aligned using the *ClustalW* program [16], to obtain the multiple sequence alignment shown in figure 1A. The three-dimensional structure is also available for 6 out of the 18 conotoxins in the alignment, namely ω-conotoxin GVIA, ω-conotoxin GVIA (O10K), ω-conotoxin MVIIC, ω-conotoxin MVIIA, ω-conotoxin So3 and the ω-conotoxin TxVII (PDB codes: 2CCO, 1TR6, 1OMN, 1OMG, 1FYG, 1F3K), thus facilitating the analysis of sequence/structure relationships.

**Figure 2:**
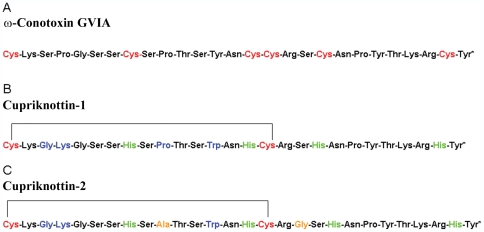
Amino acid sequence of ω-conotoxin GVIA (A) and of the redesigned peptides Cupricyclin-1 (B) and Cupricyclin-2 (C), see below.

The multiple alignment was then analysed using the Weblogo program [17] to obtain the graphic representation shown in figure 1B, from which a consensus sequence of the ω-conotoxins was derived by choosing in each position of the sequence the most frequent amino acid. The consensus sequence shown in figure 1B was the starting point for the design. However, this sequence was subsequently modified taking into account the following constraints: 1) the need to limit the hydrophobicity of the designed peptide in order to avoid aggregation phenomena (this is why Ala6, Met12, Gly25 and Gly28 where changed into Ser, Ser, Lys and Tyr residues, the second most represented residues in those positions according to the weblogo plot); 2) the need to introduce a Trp probe in the designed peptide in order to measure the copper binding affinity of the peptide using fluorescence quenching techniques (this is why Tyr13 of the consensus sequence was substituted by a Trp residue); 3) the need to constrain the intercysteines loops and the C-terminal region of the peptide so as to favour correct positioning of the His residues to generate the copper binding site (this is why Lys10 and Arg22 were both changed to Pro residues). With these changes to the consensus sequence, the designed peptide accidentaly resulted to have a high sequence similarity to our starting bait ω-conotoxin GVIA (Figure 2). Thus, ω-conotoxin GVIA three-dimensional structure was used to construct *in silico* the structural model of the first mini metalloprotein variant (see Materials and Methods section for details). One of the main problems connected with the folding of conotoxins is the high probability of formation of multiple disulphide bonds isomers of the mature peptides. It is well known in fact that *in vivo*, folding of conotoxins occurs at the prepropeptide stage (when the mature toxin sequence has two additional sequence stretches at the N-terminus) and is aided by protein disulphide isomerases [18]. In our case, to ensure that the redesigned peptide folded in a unique structure, we substituted four of the six cysteine residues of ω-conotoxin GVIA with His residues, after verifying that the interatomic distances of the introduced His residues matched those observed in the Cu,Zn superoxide dismutase copper site [19], taken as a template.

In particular, His residues were introduced in the place of Cys8, Cys15, Cys19 and Cys26. These substitutions determine the formation of a single disulphide bridge in the peptide structure, avoiding problems due to the incorrect pairing of cysteine residues, and hence favouring a unique folding of the redesigned peptide. At the same time, binding of the metal to the four His ligands was expected to constrain the polypeptide chain in a manner similar to the two missing knotted disulphides. Furthermore, binding of copper in a solvent shielded part of the molecule was expected to facilitate the achievement of a higher superoxide dismutation rate as demonstrated in Cu,Zn superoxide dismutase [20]. In fact, the electrostatic steering effect of copper on the superoxide anion substrate is enhanced if the copper charge is not screened by the solvent [20].

The redesigned miniprotein, whose amino acid sequence is shown in figure 2, has been named Cupricyclin-1 to emphasize the presence of a copper binding site and the cyclization of the polypeptide chain through one disulphide bond.

### Molecular dynamics simulations of Cupricyclin-1

**Figure 3:**
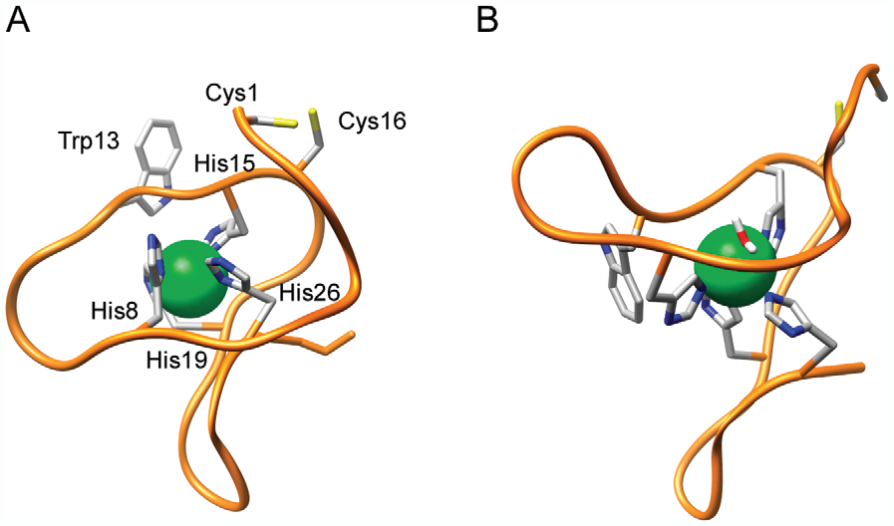
Three-dimensional model of Cupricyclin-1 before (A) and after MD simulations (B). The copper ion is represented by a green sphere. For clarity only the His ligands, Trp13, the two Cys residues and the copper-coordinating water molecule are shown. The figure was prepared using UCSF Chimera [53].

The structural model of Cupricyclin-1 was subjected to a 10 ns molecular dynamics (MD) simulation run in explicit solvent to test the stability of the introduced copper site and of the overall structure (Figure 3). In detail, the solvated molecule was first energy-minimized applying position restraints to all non-hydrogen atoms. In this phase, the distances between the copper ion and the Nε2 atoms of the four coordinating His residues were restrained to a value of 2 Å by using a harmonic potential (see Materials and Methods section for details). Subsequently, 100 ps molecular dynamics simulation under NVT conditions, followed by 100 ps NPT simulation, with position restraints on all heavy atoms of the miniprotein and with restrained copper-ligand binding distances, were performed in order to equilibrate the temperature and pressure of the system. In the following step the structure was subjected to a 5 ns MD simulation with harmonic potentials applied only to the distances between copper and its ligands, and finally to a 10 ns MD simulation without any restraint. MD simulations without restraints indicated that the redesigned peptide can stably bind copper while preserving a well defined structure. Detailed analysis of the position of the copper ion with respect to the protein atoms of Cupricyclin-1 revealed that the distances between copper and the nitrogen atoms of the four ligands ranged from 2.0 to 2.5 Å over the entire course of the production run. Moreover, two additional ligands entered the copper coordination sphere during the simulation: one water molecule (metal-ligand distance 1.8–2.2 Å) and the carbonyl oxygen of Trp13 (metal-ligand distance 2.0–2.5 Å). The coordination geometry of the copper ion remained octahedral during the simulation time with an average angle between copper and each pair of adjacent ligands of approx. 90° (values ranging from 88° to 93°). The Nε2 atoms of His8, His15, His26 and the carbonyl oxygen of Trp13 lie in one plane, while the Nε2 atom of His19 and the water molecule are above and below the plane. This coordination geometry is similar to that observed in Cu,Zn superoxide dismutase (See Figure S1); indeed, a copper-coordinating water molecule is also present in this enzyme [19].

### Cupricyclin-1 synthesis and characterization

Cupricyclin-1 was synthesised by standard Fmoc chemistry, loaded on a Vydac C_18_ semipreparative column and isolated from by-products by HPLC. The major peptide-containing fraction was then air oxidized at 0.01% concentration (w/w) in 0.1 M NH_4_HCO_3_ to allow formation of the single disulphide bond. The cyclic miniprotein was finally purified from dimers/multimers by HPLC connected with an ESI-IT mass spectrometer and eluates were collected and characterized by MALDI-TOF analysis.

The MALDI-TOF spectrum of Cupricyclin-1 shows a main peak with a mass of 3154,00 a.m.u., corresponding to Cupricyclin-1 in the cyclic oxidized form (See Figure S2A). To confirm this result Cupricyclin-1 was treated either with the alkylating agent iodoacetamide (IAM), or with dithiothreitol (DTT), or else with both DTT and IAM. In the case of IAM treatment no modification was observed in the MALDI-TOF spectrum profile (Figure S2B), while the sample treated with DTT showed, as expected, an increase of 2 a.m.u. of the main MALDI-TOF spectrum peak, consistent with reduction of the two Cys residues (Figure S2C). Finally, treatment with DTT followed by IAM yielded a main peak centered at 3270 a.m.u., corresponding to the alkylation of two Cys residues (mass increase +116 a.m.u.) (Figure S2D).

### Cupricyclin-1 metal binding ability

**Figure 4:**
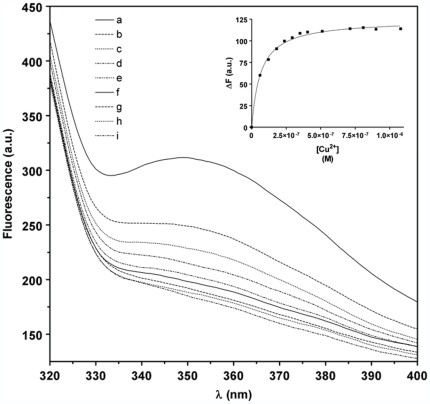
Emission fluorescence spectra of Cupricyclin-1 in the presence of increasing amounts of Cu^2+^ ions. (a) apo Cupricyclin-1 (0.5 µM); (b) a+CuCl_2_ 0.06 µM; (c) a+CuCl_2_ 0.12 µM; (d) a+CuCl_2_ 0.18 µM; (e) a+CuCl_2_ 0.24 µM; (f) a+CuCl_2_ 0.30 µM; (g) a+CuCl_2_ 0.36 µM; (h) a+CuCl_2_ 0.41 µM and (i) a+CuCl_2_ 0.51 µM (only the spectra relative to a stoichiometric ratio Cu^2+^:Cupricyclin-1 ≤1 are reported); **(inset)**: Determination of the dissociation constant of Cu^2+^ to Cupricyclin-1 by fluorescence quenching experiments. The non linear fitting curve of fluorescence decrease at 352 nm as function of increasing amounts of Cu^2+^ ions is shown as a continuous line.

Evidence of Cu^2+^ binding to Cupricyclin-1 was first obtained by fluorescence quenching experiments, exploiting the ability of copper ions to quench the fluorescence emitted by the single Trp residue (Trp13) upon binding to the miniprotein [5], [21]. Fluorescence quenching was dependent on metal concentration and reached a maximum at a [Cu^2+^]/[peptide] of approx. 1∶1 (Figure 4). These characteristics are consistent with an energy-transfer mechanism from the tryptophan residue to the copper bound at the binding site. Fluorescence quenching due to copper binding to Cupricyclin-1 was used to determine the binding affinity of the miniprotein for Cu^2+^. The fluorescence intensity decrease at 352 nm as a function of increasing amounts of copper ions was fitted with a non linear regression curve (Figure 4), obtaining a copper dissociation constant of 3.8 (±1.4)×10^−8^ M.

**Figure 5:**
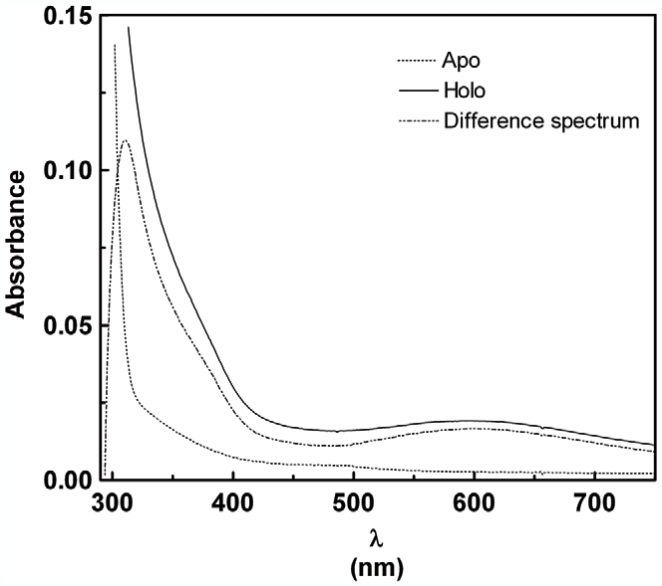
Optical spectra of apo and holo Cupricyclin-1 (0.6 mM in 50 mM sodium acetate buffer, pH 6.5). The difference spectrum is also shown to evidence the appearance of a band at 300–312 nm, indicative of a Cu^2+^-histidine charge-transfer [21], [22].

Further evidence of copper-peptide binding was obtained by optical spectroscopy analysis. Addition of a stoichiometric amount of CuCl_2_ to Cupricyclin-1 (0.6 mM final concentration) induced the appearance of absorption bands with maxima at 300–312 nm and 520–600 nm (Figure 5), the first indicative of a Cu^2+^-histidine charge-transfer [22], the second due to electronic transitions of copper *d-d* orbitals and typical of copper complexes with nitrogen ligands [22], [23]. The calculated molar extinction coefficient of holo-Cupricyclin-1 at 595 nm is 203 M^−1^ cm^−1^.

The stability of the copper site of Cupricyclin-1 was also investigated by differential scanning calorimetry (DSC). The DSC trace of Cupricyclin-1 with bound copper exhibited a strong exotherm beginning above 55°C and showing a minimum at 95°C. The exotherm disappeared after copper removal (see Figure S3). No clear endotherm was evident in the thermograms, due to the fact that the peptide almost completely lacks defined structural elements which can yield DSC signals (such as hydrogen bonds). In copper proteins such as azurin and plastocyanin, the exotherm is attributed to copper-dependent redox reactions with cysteines following copper release [24]–[26]. By analogy, DSC results indicate that the metal site of Cupricyclin-1 is stable up to 55°C and that copper release is complete only at 95°C (the T_1/2_ for copper release being approx. 75°C). The stability of the copper site in Cupricyclin-1 well compares with that of natural metalloproteins such as azurin and plastocyanin which display complete copper release at temperature values ranging from 70 to 85°C depending on the metal oxidation state and the DSC scanning rate [24]–[26].

The fluorescent binding titration data suggested a copper∶peptide stoichiometry of 1∶1. However this result would also be compatible with the presence of higher cross-linked oligomers where the copper∶peptide ratio is 1∶1 as well. To rule out this possibility a MALDI-TOF spectrum of Cupricyclin-1 was recorded, showing that only the apo and holo monomeric forms of the peptide were present in the sample (see Figure S4).

### EPR spectroscopy characterization of Cupricyclin-1

**Figure 6:**
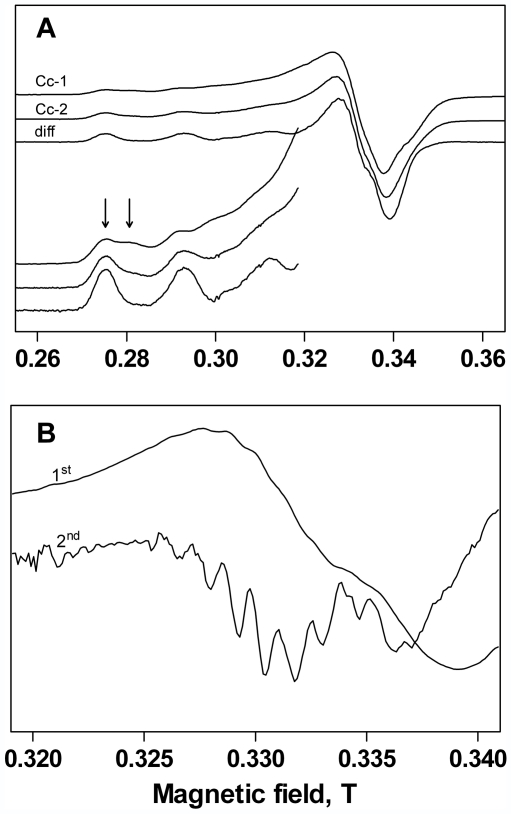
EPR spectrum of Cupricyclins. **Panel A shows the spectra of Cupricyclin-1 (Cc-1), Cupricyclin-2 (Cc-2) and a difference spectrum (diff) obtained by arbitrarily subtracting a fraction of Cc-1 from Cc-2.** Arrows on the first hyperfine of Cc-1 reveals the signal heterogeneity. Experimental details in the text. Panel B displays a detail of the perpendicular region of the difference spectrum, shown both as the standard first derivative lineshape and as the second derivative curve, to better evidence the superhyperfine lines due to interaction of copper with the four nitrogen nuclei of the coordinating histidine residues.

The liquid nitrogen EPR spectrum of Cupricyclin-1 (Fig. 6A, spectrum “Cc-1”) appeared to arise from two spectroscopically distinguishable species of copper, as can be clearly seen by the split peak of the low magnetic field hyperfine line (arrows). The EPR parameters for a copper complex are determined by the chemical composition and the physical constraints on the atoms nearest to the metal ion, with g and A values strictly depending on the composition of the ligand atoms bound to the copper. The EPR spectrum of Cupricyclin-1, although heterogeneous, reveals an essentially axial g tensor, with g_//_ values in the range 2.21–2.25. The copper hyperfine values A_//_, on the other hand, can be estimated to be above 0.018 cm^−1^. These values are typical of a type-2 mono Cu(II) complex, *i.e.* a square planar ligation of the copper ion with possible additional axial ligations [27]. The empirical quotient *f* = g_//_/|A_//_| is considered an index of tetrahedral distortion, ranging from 105 to 135 for square-planar copper structures [28]. In this respect, the EPR parameters of Cupricyclin-1 suggest a low degree of tetrahedral distortion. Also worth of note is the absence of an anomalous number of lines in the parallel region and of any half-field signal arising from a triplet spin state, strongly supporting the hypothesis that the peptide is monomeric in solution. All these data are fully consistent with the coordination environment predicted by MD simulations. Variable power experiments in the 2.5–190 mW range (not shown) did not discriminate between the two signals, which had similar saturation behavior, suggesting that the spectroscopic inhomogeneity could arise from slightly different conformational states in a single copper coordination environment.

Peisach and Blumberg showed that the g and A values in the parallel region of the EPR spectrum of copper sites give information on the type of ligating atoms [27]. According to their method, the parameters of Cupricyclin-1 are in agreement with a 4-nitrogen coordination sphere. This is also consistent with the nitrogen-induced superhyperfine splitting pattern observed in the closely related Cupricyclin-2 (see below).

The metal-binding region of native SOD constitutes the catalytic active site, therefore it is interesting to study in detail the metal sites of SOD-mimics in order to correlate the structure to the biological behaviour. In the native enzyme, copper is coordinated by three imidazole residues and a water molecule, while an imidazolate bridge links the copper and zinc sites. This results in a distorted tetrahedral structure of the copper site that can be readily inferred from its strong anisotropic EPR signal [29] with an *f* value of 159 for bovine Cu,Zn SOD [30]. The site is very flexible, switching from an irregular pyramid containing one weak axial water molecule in the oxidized form to a tricoordinated conformation in its reduced form. This is a crucial aspect in that high SOD activity is related to high flexibility in the conformation around copper [31], [32]. In this respect, the copper environment in Cupricyclin-1 seems to be significantly more rigid, accounting for its SOD activity that, although significant for a SOD-mimic (see below), is still far from that of the native enzyme. It should also be reminded that a five-coordinated square pyramidal or trigonal bipyramidal structure is more favourable for a good SOD activity than a four-coordinated square planar structure [33].

### NMR spectroscopy characterization of Cupricyclin-1

**Figure 7:**
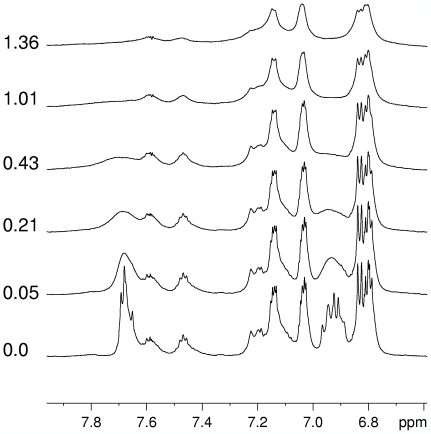
Titration of Cupricyclin-1 with CuSO_4_ monitored by ^1^H NMR. The molar ratio CuSO_4_/Cupricyclin-1 is reported on the left side of each spectrum.

Copper interaction with Cupricyclin-1 was also investigated by NMR spectroscopy. The assignment of the ^1^H spectrum of apo-Cupricyclin-1 is shown in Table 1. Copper interaction with apo-Cupricyclin-1 was studied by addition of aliquots of CuSO_4_ to a 1.06×10^−3^ M solution of the peptide in H_2_O/D_2_O (9∶1 v/v). The first CuSO_4_ addition (Cu^2+^/peptide ratio 0.05∶1) caused a drastic broadening of Hδ (between 7.0 and 6.9 ppm) and Hε proton signals (between 7.7 and 7.6 ppm) of the imidazole rings of all four histidine residues (see Figure 7). On the other hand, the signals of Trp, Tyr and of other amino acid residues remained unchanged. Stepwise addition of CuSO_4_ led to a further broadening of His signals and to a decrease of the intensity and only minor broadening of other ^1^H amino acid signals (Figure 7). At a Cu^2+^/peptide ratio higher than 1.0 a further broadening of all ^1^H amino acid signals was observed.

**Table 1:** Assignments of ^1^H and ^13^C resonances of apo-Cupriknottin 1 in D_2_O at 300 K, pH 7.0.

Amino acid	Atom	Chemical shift (ppm)				
		Cα	Cβ,β′	Cγ,γ′	Cδ	Others
Asn 20	^1^H	4.88	2.83; 2.62			
	^13^C	49.6	37.8			
Asn 14	^1^H	4.50	2.60			
	^13^C	52.7	37.2			
Arg a	^1^H	4.33	1.83; 1.74	1.58	3.12	
	^13^C	55.2	29.4	25.8	42.0	
Arg b	^1^H	4.26	1.65	1.49; 1.43	3.07	
	^13^C	54.6	29.8	25.7	42.0	
Gly 3, 5	^1^H	3.95				
	^13^C	44.1				
His a	^1^H	4.62	3.10; 3.01		6.93	
	^13^C	55.3	30.0		118.8	
His b	^1^H	4.52	3.08		6.91	
	^13^C	55.8	29.6		118.8	
His c	^1^H	4.58	3.04; 2.97		6.89	
	^13^C	55.1	30.0		118.8	
His d	^1^H	4.58	3.04; 2.98		6.87	
	^13^C	55.1	30.0		118.8	
Lys 2, 4, 24	^1^H	4.25; 4.24; 4.19	1.64	1.36		2.94
	^13^C	55.0; 55.5; 55.6	27.8	23.4		40.8
Pro 10	^1^H	4.35	2.26; 1.91	1.96	3.70	
	^13^C	62.6	30.8	26.1	49.4	
Pro 21	^1^H	4.32	2.13; 1.65	1.85; 1.63	3.59	
	^13^C	62.2	30.6	25.5	49.3	
Ser 6, 7, 9, 12, 18	^1^H	4.35; 4.41; 4.48	3.82			
	^13^C	57.8;57.3; 57.1	62.4			
Thr a	^1^H	4.28	4.19	1.13		
	^13^C	60.8	68.2	20.3		
Thr b	^1^H	4.25	4.15	1.17		
	^13^C	60.6	68.5	20.3		
Trp 13	^1^H	4.66	3.26; 3.21		7.19	7.56 (Hε); 7.21 (Hζ3); 7.12 (Hη); 7.46 (Hζ2)
	^13^C	56.2	28.1		125.8	119.7 (Cε); 123.4 (Cζ3); 120.7 (Cη); 113.4 (Cζ2)
Tyr 22	^1^H	4.58	3.11; 2.96		7.12	6.81 (Hε)
	^13^C	56.7	36.9		131.8	117.1 (Cε)
Tyr 27	^1^H	4.35	3.03; 2.87		7.02	6.78 (Hε)
	^13^C	56.7	38.1		132.0	116.9 (Cε)
Cys a	^1^H	4.61	3.18; 2.98			
	^13^C	54.5	39.3			
Cys b	^1^H		3.13			
	^13^C		41.0			

Letters a, b, c, d in the first column indicate the amino acid residues whose specific position in the peptide chains was not assigned.

These results suggest that at low Cu^2+^/peptide ratios (≤1), a peptide–Cu^2+^ complex is formed with a specific interaction between the metal and all the four His residues of the peptide, as indicated by the broadening of histidine signals. At high Cu^2+^/peptide ratios (>1), when the specific binding is already saturated, a nonspecific interaction between Cu^2+^ and other amino acid residues is manifested by a further, minor, intensity decrease and broadening of all the amino acid signals, as already observed in a previous study from our lab on a structurally unrelated copper-binding peptide [5].

### Determination of superoxide dismutase activity of Cupricyclin-1

The ability of the copper ion bound to Cupricyclin-1 to be reversibly reduced, a prerequisite for any redox-mediated catalytic activity, was tested by assaying the superoxide dismutase activity of Cupricyclin-1 using the pyrogallol enzymatic assay [34]. From the concentration of superoxide dismutase and Cupricyclin-1 required to reach 50% inhibition of the reaction rate of pyrogallol autoxidation (2.7×10^−9^ M and 4.7×10^−5^ M, respectively, Table 2) and taking as a reference the enzymatic activity of superoxide dismutase (3.9×10^9^ M^−1^ s^−1^) [35], a superoxide dismutation rate of 1.8×10^5^ M^−1^ s^−1^ was calculated for Cupricyclin-1, a value of the same order of magnitude of that observed for Cupryphans [5] and for non peptidic superoxide dismutase mimics [36].

**Table:** **Table 2.** Superoxide dismutase activity of Cupriknottins.

	[SOD]	[Cupryphan]	[Cupriknottin-1]	[Cupriknottin-2]
50% inhibition*K_1_, K_2_*	2.7×10^−9^ M3.9×10^9^ M^−1^ s^−1^	8.5×10^−5^ M1.0×10^5^ M^−1^ s^−1^	4.7×10^−5^ M1.8×10^5^ M^−1^ s^−1^	3.1×10^−5^ M3.4×10^5^ M^−1^ s^−1^

### Design and characterization of Cupricyclin-2

To study the role of peptide backbone flexibility in the metal binding affinity and redox properties of Cupricyclins, an additional variant was designed in which the intercysteines loop residue Pro10 was substituted with an Ala residue and a Gly residue was introduced between Arg17 and Ser18 to increase the length of the loop connecting His15 to His19 (Figure 2C). The structural properties of the novel peptide, named Cupricyclin-2, were studied through a combination of MD simulations, fluorescence, optical and EPR spectroscopy, and its superoxide dismutase activity was determined. Molecular dynamics simulations, carried out following the same protocol used for Cupricyclin-1, indicated that Cupricyclin-2 can stably bind copper in a similar geometry as that observed for Cupricyclin-1. Also in the case of Cupricyclin-2 the coordination geometry of the copper ion remains octahedral during the time of the simulations, with the nitrogen atoms of His8, His15 and His20 together with the carbonyl oxygen of Gly18 lying in one plane and the nitrogen atom of His27 and a water oxygen acting as apical ligands.

Comparative analysis of the MD simulations trajectories of Cupricyclin-1 and Cupricyclin-2 indicated that, indeed, substitution Pro10Ala and insertion of Gly18 changed the conformational stability and flexibility of the molecule as well as the pattern of hydrogen bond interactions and of copper-protein interactions. A decrease of the magnitude of conformational changes at the beginning of the simulation, a higher flexibility over the course of the production run as well as a decrease of the number of hydrogen bonds of Cupricyclin-2 compared to Cupricyclin-1 were observed.

Despite the similarity of the starting conformations, the distribution of RMSD values as a function of time shows that, initially, Cupricyclin-1 underwent bigger conformational changes than Cupricyclin-2 with respect to the starting conformation (see Figure S5, top panel). However, analysis of the RMSD plots indicates that, after the initial conformational changes occurred during the equilibration period, Cupricyclin-1 remained more stable than Cupricyclin-2 over the course of the production run (lower fluctuations of the RMSD values; Figure S5, top panel). It is noticeable in the RMSD plot that Cupricyclin-2 underwent a reversible structural change during the period from 4.7 to 5.7 ns of the unrestrained simulation.

Analysis of the matrix of Cα RMSD values of every frame with respect to every other frame of the production run simulation (a measure of the extent of conformational variability observed in the system) shows that Cupricyclin-1 was represented by two distinct clusters of conformations (see Figure S5, bottom panel A). A clear structural rearrangement at around 4 ns of the unrestrained run visible on the matrix representation coincides with a very small increase of the values on the RMSD plot (Figure S5, top panel). Analysis of the RMSD matrix of Cupricyclin-2 suggests that the protein underwent several reversible transitions during the simulation. Moreover, a higher similarity (lower RMSD values) between the members of the same clusters of Cupricyclin-1 than within the clusters of Cupricyclin-2 is observed (Figure S5, bottom panel B).

From fluorescence quenching experiments a copper dissociation constant of 10.7 (±1.1)×10^−8^ M was determined for Cupricyclin-2, suggesting that a higher backbone flexibility causes a slight decrease of the peptide affinity for copper ions. Optical spectroscopy studies demonstrated that, also in the case of Cupricyclin-2, addition of a stoichiometric amount of CuCl_2_ induces the appearance of absorption bands with maxima at 300–312 nm and 520–600 nm which, by analogy with Cupricyclin-1, were attributed to a Cu^2+^-histidine charge-transfer band and to electronic transitions of copper *d-d* orbitals typical of copper complexes with nitrogen ligands, respectively [22], [23]. The calculated molar extinction coefficient at 525 nm was 476 M^−1^ cm^−1^.

Quite interestingly, the EPR spectrum of Cupricyclin-2 was much less heterogeneous than that of Cupricyclin-1 (Fig. 6A, “Cc-2” vs “Cc-1”). However, both spectra appeared to be constituted by the same species, although with different fractional contributions. As a matter of fact, a “pure” EPR lineshape was readily obtained by subtracting a fraction of the Cupricyclin-1 lineshape from the spectrum of Cupricyclin-2 (Fig. 6A, “diff”). The difference spectrum clearly showed at least nine superhyperfine lines in the perpendicular region of the spectrum (Fig. 6B), with position and average coupling constant (≈14 G) typical of copper(II) complexes with a minimum of four magnetically equivalent histidine residues on the coordination plane, this result being fully consistent with the body of our data. Again, the EPR parameters were within a four nitrogens coordination sphere, according to the Peisach-Blumberg plot [27].

Finally, the superoxide dismutase activity of Cupricyclin-2 was found to be slightly higher than that of Cupricyclin-1, the reaction rate being 3.4×10^5^ M^−1^ s^−1^ as compared to 1.8×10^5^ M^−1^ s^−1^ determined for Cupricyclin-1 (Table 2). This result suggests that, while a higher flexibility of the peptide backbone decreases the stability of the copper site, the same flexibility may facilitate coordination of both Cu^2+^ and Cu^1+^ within the same binding site with positive effects on the copper reduction rate and thus on the ability of the mini metalloprotein to act as a catalyst in redox reactions.

### Conclusions

The use of metal-binding peptides in green chemistry applications and bioremediation is becoming an increasingly adopted strategy [37]. This is due to the fact that small stable scaffolds can be engineered to host a metal binding site starting from basic structural principles [1]. On the other hand, the insertion of metal binding residues can lead to a destabilization of the scaffold and to undesired structural changes [1]. Knottins represent one of the most stable scaffolds, being constrained by three disulphide bonds, and are extremely tolerant to sequence variation thus representing optimal candidates for redesign strategies [4]. However the presence of three disulphide bonds implies that the attainment of a unique structure during folding is severely hampered by the possible formation of multiple disulphide bonds isomers with a consequent low yield of the desired structural form [18]. To overcome this problem, in this work we have adopted the strategy of substituting the two knotted disulphides of a member of the knottins family, ω-conotoxin GVIA, with a metal binding center. In this way it has been possible to obtain a rigid metal-coordination environment while at the same time avoiding the problems intrinsically connected with the presence of multiple disulphide bonds. The redesigned peptides Cupricyclin-1 and -2 bind copper ions with a fairly high affinity and display reversible reduction of the copper center, catalyzing the dismutation of superoxide anions at an acceptable rate, compared to other superoxide dismutase mimics [5], [36]. One way of improving the catalytic efficiency of Cupricyclins would be that of mimicking the electrostatic potential distribution observed in Cu,Zn superoxide dismutases in which a positively charged active site is hosted in an essentially negatively charged protein surface [38]. This gives rise to an electrostatic steering effect which makes productive any collision of the substrate with the protein surface. In fact the negatively charged substrate is repulsed by the negatively charged protein surface and attracted by the positively charged active site [38]. In this regard, it must be noted that the C-terminal basic residues Lys24 and Arg25 of Cupricyclin-1 (Lys25 and Arg26 of Cupricyclin-2), according to the structural models obtained by MD simulations, are located on the opposite side of the copper solvent accessible site (Figure 3). These two residues could be substituted by negatively charged ones thus increasing the asymmetry of the electroststic potential distribution of the two peptides and their electrostatic steering effect on the substrate.

An additional issue that emerges from the results presented in this work is the difficulty in reconciling structural rigidity with the flexibility required to bind a metal ion in two different valence states. As stated in the Results and Discussion section, the imidazolate bridge that links the Cu and Zn ions in Cu,Zn SOD results in a distorted tetrahedral structure of the copper site which is crucial for high SOD activity [31], [32]. The same geometry cannot be reproduced in our design making the copper environment in Cupricyclins significantly more rigid and resulting in a relatively low SOD activity. From this viewpoint, it is known that mononuclear metal centers based on iron, manganese and nickel display superoxide dismutase activity [39]. MnSOD, in particular, in which the metal ion is coordinated by one Asp and three His residues [39] appears to be a good model for future developments of our design. Further studies will be required to address this point and obtain high affinity metal binding peptides endowed with faster redox kinetics.

## Materials and Methods

### Molecular modelling

Initial structural models of Cupricyclin-1 and Cupricyclin-2 were built using the average solution structure of ω-conotoxin GVIA as a template (PDB code 2CCO [14]). The conformation of the inserted His residues was chosen using the standard Deep View rotamer library [40] so as to form a tetragonal metal binding site with geometry and ligand distances comparable to the copper binding site of bovine Cu,Zn superoxide dismutase (PDB code 2SOD [19]).

### Molecular dynamics simulations

All simulations were performed with the GROMACS (v. 4.0.7) simulation package [41] together with the Amber99SB force field [42]. The parameter sets were generated by the *tleap* program of the Amber (v.9) molecular dynamics package [43]. The conversion into the GROMACS topology was performed by the Perl script *amb2gmx.pl* [44]. Lennard-Jones parameters (R_min_/2 = 1.0330 Å, ε = 0.0427 kcal/mol) published by C. S. Babu and C. Lim were chosen for the copper ion [45].

Copper-coordinating residues (His8, His15, His19, His26 of Cupricyclin-1, and His8, His15, His20, His27 of Cupricyclin-2) were protonated at the Nδ1 position. Each molecule was placed in a rhombic dodecahedron box. The size of the simulation boxes was chosen to satisfy the condition that the distance between any protein atom and the box boundaries was larger than 1 nm. The simulation boxes were solvated with SPC/E [46] water molecules and neutralized with Cl^−^ ions. The solvated molecules were energy minimized by using the Steepest Descent algorithm until the convergence criterion of 100 kJ mol^−1^ nm^−1^ was reached (approx. 4000 steps for Cupricyclin-1 and 5100 steps for Cupricyclin-2). During the minimization runs position restraints of 1,000 kJ mol^−1^ nm^−2^ were applied to all non-hydrogen atoms of the proteins, except for copper-binding residues. The distances between the copper ion and the Nε2 atoms of the 4 coordinating histidine residues of each molecule were restrained to a value of 0.2 nm by using a harmonic potential of 100,000 kJ mol^−1^ nm^−2^ (bond type 6 of the GROMACS topology). 100 ps molecular dynamics simulation under NVT conditions, followed by 100 ps NPT simulation, with position restraints on all heavy atoms of the proteins and with restrained copper-ligand binding distances, were performed in order to equilibrate the temperature and pressure values of the systems. In the following two steps the structures were submitted to a 5 ns molecular dynamics simulation with harmonic potentials applied only to the distances between copper and its ligands and finally to a 10 ns simulation without any restraint.

A constant temperature value of 298 K was maintained during the simulations with the V-rescale algorithm [47], with a coupling constant of 0.1 ps. Parrinello-Rahman barostat [48] with a coupling constant of 2.0 ps was used for pressure coupling. The simulations were performed with a time step of 2 fs. Lennard-Jones interactions were cut off at 1.0 nm. Particle-mesh Ewald method (PME) [49] was applied to electrostatic interactions with a real space cutoff of 1.0 nm. All bonds, except those of water molecules, were constrained with the LINCS algorithm [50]. The SETTLE algorithm [51] was used for water molecules.

### Peptides synthesis

Cupricyclins were synthesised by standard Fmoc chemistry on an automated Peptide Synthesizer (Pioneer, Applied Biosystems). The protected peptides were grown on a PAL-resin with a high level of modification, using the HATU/DIPEA amino acid activation, according to manufacturer's instructions. Side chain protection scheme was the following: Asp(*Ot*-Bu), Cys(Trt), Trp(Boc) and Lys(*t*-Boc). After synthesis, both peptides were released and deprotected by treatment with TFA/H_2_O/triisopropylsilane (90∶5∶5 by volume) for 1 hr at room temperature. The cleavage mixture was filtered under vacuum into *t*-butyl-methyl ether at −20°C. Peptides were collected by centrifugation at 2500 rpm for 20 min at 4°C and washed with *t*-butyl-methyl ether. The pellet was dissolved in 50% acetonitrile and lyophilized. Peptides were loaded on a Vydac C_18_ semipreparative column (10 mm×250 mm, 5 µm particle size, 300 Å pore size) and isolated from by-products with an HPLC apparatus (model K1001, Knauer GmbH, Berlin, Germany), using a linear gradient of 5–80% acetonitrile, containing 0.2% TFA, in 60 min. Elution was monitored by absorbance at 230 nm with an on-line UV detector (model K2501, Knauer GmbH, Berlin, Germany) and peptide fractions were manually collected. The major peptide-containing fractions were air oxidized at 0.01% concentration (w/w) by magnetic stirring in 0.1 M NH_4_HCO_3_ for 30 min at room temperature. The cyclic peptides were finally purified from dimers/multimers by HPLC, as described for purification of synthetic products (see above), and characterized by infusion in an electrospray mass spectrometry (ES-IT, mod. LCQ, ThermoElectron, San Jose, CA, USA).

### Fluorescence quenching experiments

Fluorescence spectra of Cupricyclins were recorded at room temperarure with a Jasco FP-6200 spectrofluorometer. The excitation wavelength was 280 nm and the excitation and emission slits were set at 10 and 5 nm, respectively. Emission spectra were recorded in the 320 to 400 nm range. Peptides were dissolved at a concentration of 4.9×10^−7^ M in 50 mM sodium acetate buffer (pH 6.5). For copper binding studies, CuCl_2_ (6.2×10^−6^ M) was added in aliquots and fluorescence was measured after 5 min after addition. The dissociation constant values given in the text are the average of at least three independent experiments.

### Optical spectroscopy

UV-Vis electronic absorption spectra were recorded on an Perkin-Elmer λ14P spectrophotometer. Protein concentration was determined by UV absorption at 280 nm, by using a molar extinction coefficient (ε_280_) of 8605 M^−1^ cm^−1^ [52].

### EPR spectroscopy

Low temperature X-band EPR spectra were recorded on a Bruker ECS 106 spectrometer equipped with an ER4111VT temperature controller. In detail, spectra were recorded at 9.556 GHz and 115 K. Cupricyclins concentration was 0.5 mM in 50 mM sodium acetate buffer, pH 6.5.

### NMR spectroscopy

NMR spectra were recorded at 300 K on a Bruker AVANCE AQS 600 spectrometer operating at 600.13 MHz and equipped with a Bruker multinuclear z-gradient probehead. Peptide solution was prepared in H_2_O with 10% of D_2_O (v/v). pH values of Cupricyclin-1 solution was adjusted to 8.0 by the addition of 0.1 M NaOH in D_2_O to deprotonate the N-H/N-D group of the imidazole ring of histidine residues. In all the ^1^H spectra, a soft presaturation of the HOD residual signal was applied.

### Superoxide dismutase activity assay

Superoxide dismutase activity of Cupricyclins was determined using the pyrogallol enzymatic assay [34]. The assay was carried out at 30°C, in 20 mM Tris-HCl buffer pH 8.2, containing 1 mM EDTA and 200 µM pyrogallol, recording the absorption increase at 420 nm for 3 min. Bovine erythrocytes Cu,Zn superoxide dismutase (0.1 µM in 20 mM Tris HCl buffer pH 8.2) was used as a standard.

## Supporting Information

Figure S1 **Copper coordination environment in Cupricyclin-1 and Cu,Zn superoxide dismutase.**
(DOC)

Figure S2 **MALDI-TOF characterization of apo-Cupricyclin-1.**
(DOC)

Figure S3 **Differential Scanning Calorimetry analysis of Cupricyclin-1.**
(DOC)

Figure S4 **MALDI-TOF spectrum of holo-Cupricyclin-1.**
(DOC)

Figure S5 **MD simulations RMSD analysis of Cuprycyclin-1 and -2.**
(DOC)
