# Supplementary material for: Cupricyclins, Novel Redox-Active Metallopeptides Based on Conotoxins Scaffold
Source: PLoS One. 2012 Feb 3;7(2):e30739. doi: 10.1371/journal.pone.0030739 (PMC3272027; doi:10.1371/journal.pone.0030739)
Supplement: Figure S1 — Copper coordination environment in Cupricyclin-1 and Cu,Zn superoxide dismutase. (DOC) [file pone.0030739.s001.doc]

Figure S1


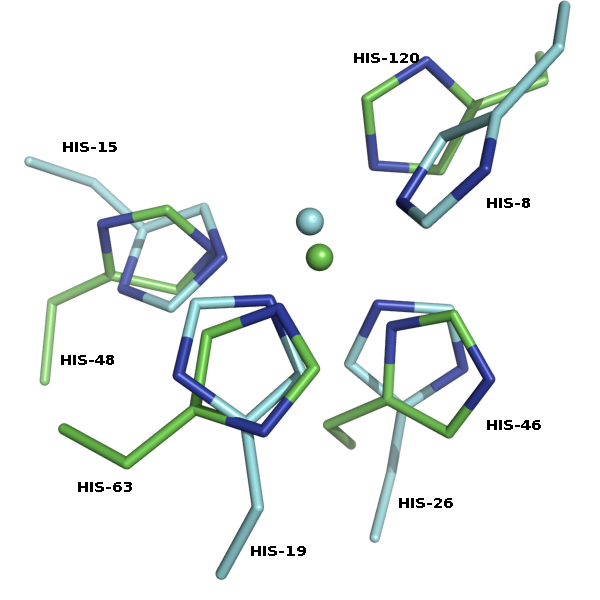


Schematic representation of the copper coordination environment in Cupriknottin-1 and bovine Cu,Zn Superoxide dismutase. Copper ion and carbon atoms of Cupriknottin-1 and superoxide dismutase are coloured in cyan and green, respectively.
