# Supplementary material for: Cupricyclins, Novel Redox-Active Metallopeptides Based on Conotoxins Scaffold
Source: PLoS One. 2012 Feb 3;7(2):e30739. doi: 10.1371/journal.pone.0030739 (PMC3272027; doi:10.1371/journal.pone.0030739)
Supplement: Figure S2 — MALDI-TOF characterization of apo-Cupricyclin-1. (DOC) [file pone.0030739.s002.doc]

Figure S2


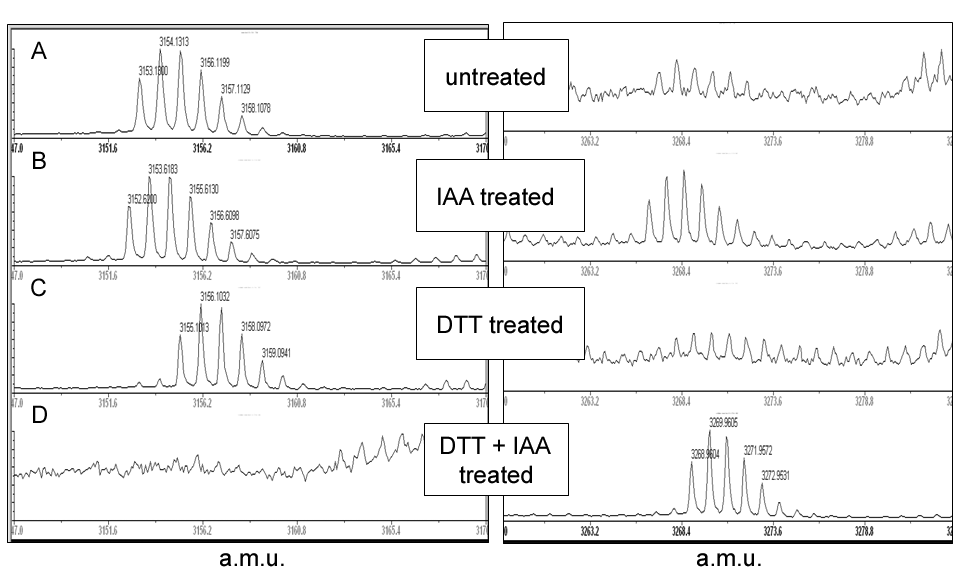


MALDI-TOF spectrum of Cupriknottin-1. A) Untreated sample; B) Sample treated with the alkylating agent iodoacetamide (IAM); C) Sample treated with dithiotreitol (DTT); D) Sample treated with DTT and IAM. Note the absence of changes in the MALDI-TOF spectrum in panel B, the increase of 2 amu of the peaks in panel C and the increase of 116 amu of the peaks in panel D.
