# Supplementary material for: Cupricyclins, Novel Redox-Active Metallopeptides Based on Conotoxins Scaffold
Source: PLoS One. 2012 Feb 3;7(2):e30739. doi: 10.1371/journal.pone.0030739 (PMC3272027; doi:10.1371/journal.pone.0030739)
Supplement: Figure S3 — Differential Scanning Calorimetry analysis of Cupricyclin-1. (DOC) [file pone.0030739.s003.doc]

**Figure S3**


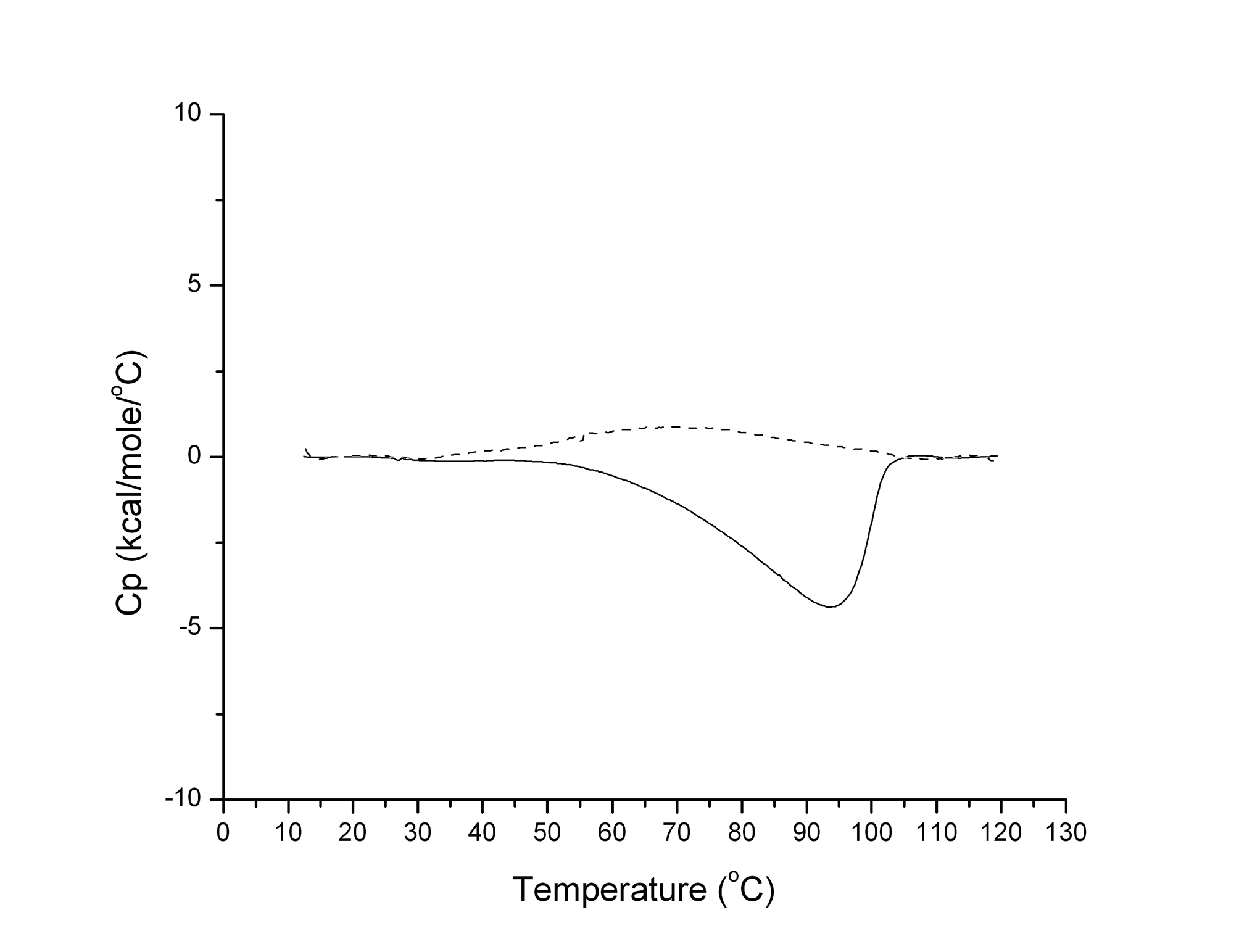


Figure S3. Differential Scanning Calorimetry analysis of Cupricyclin-1 before (continuous line) and after removal of the copper ion with Chelex resin (dashed line). DSC experiments were performed with a MicroCal VP-DSC microcalorimeter (MicroCal). The peptide was diluted in 100 mM sodium acetate buffer pH 6.5 and gently degassed before scanning. The peptide concentration was 0.12-0.25 mg/ml, and the scan speed was 60°C/h. No reversibility of thermal unfolding was observed at the end of the scan, therefore the second scan was used as a baseline to correct the thermograms. To remove copper, Cupricyclin-1 was incubated overnight with Chelex resin (Biorad) in 100 mM sodium acetate buffer pH 6.5, followed by centrifugation to recover the peptide.
