# Supplementary figures and images for: Cupricyclins, Novel Redox-Active Metallopeptides Based on Conotoxins Scaffold
Source: PLoS One. 2012 Feb 3;7(2):e30739. doi: 10.1371/journal.pone.0030739 (PMC3272027; doi:10.1371/journal.pone.0030739)

**Figure S4**


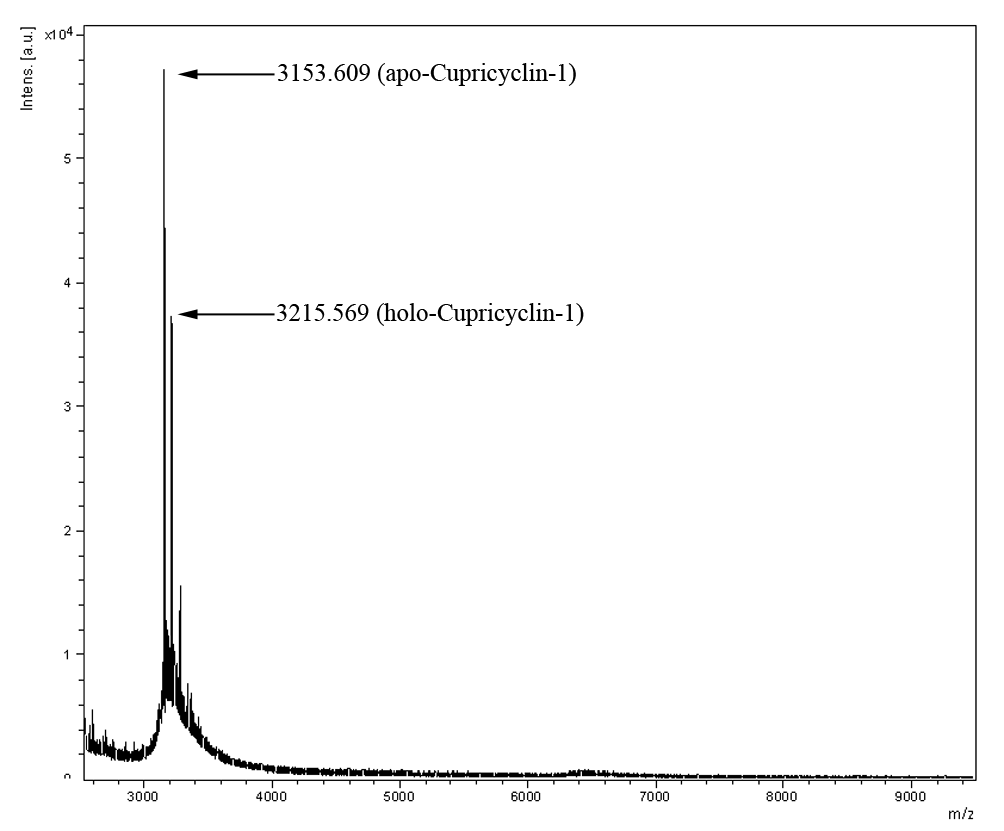


Figure S4. MALDI-TOF spectrum of Cupricyclin-1 in the 3000-9000 a.m.u region.

Supplement: Figure S4 — MALDI-TOF spectrum of holo-Cupricyclin-1. (DOC) [file pone.0030739.s004.doc]
