# Supplementary material for: Cupricyclins, Novel Redox-Active Metallopeptides Based on Conotoxins Scaffold
Source: PLoS One. 2012 Feb 3;7(2):e30739. doi: 10.1371/journal.pone.0030739 (PMC3272027; doi:10.1371/journal.pone.0030739)
Supplement: Figure S5 — MD simulations RMSD analysis of Cuprycyclin-1 and -2. (DOC) [file pone.0030739.s005.doc]

**Figure S5**


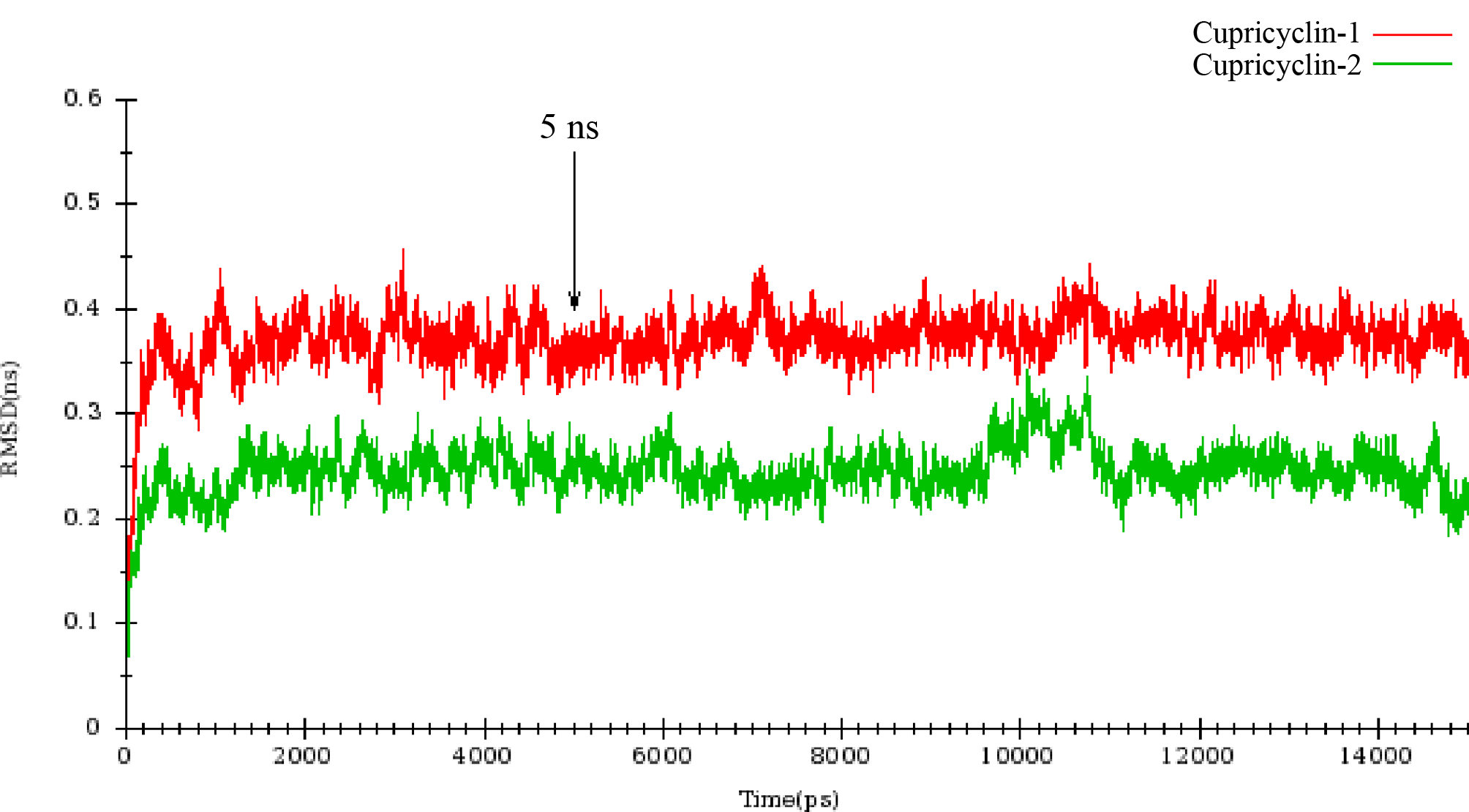


Figure S5, top panel. Cα RMSD values as a function of the MD simulation time. The final structures of the pressure equilibration simulations were chosen as the reference or “starting” structures for the analysis. The arrow indicates the moment in which the harmonic potential applied on the distances between the copper atom and the four histidine nitrogen atoms was switched off.


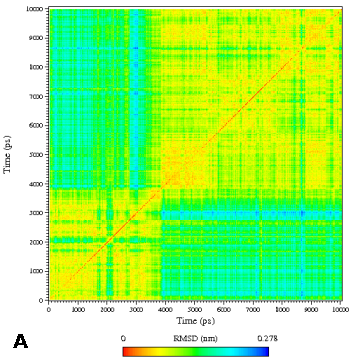

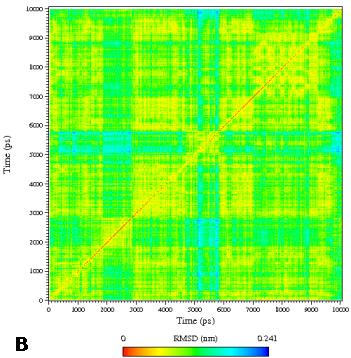


Figure S5, bottom panel. Pair-wise Cα RMSD matrix calulated for the production run with a time step of 10 ps. (A) Cupricyclin-1 (B) Cupricyclin-2. Note the two defined structure clusters in Cupricyclin-1 (low RMSD values, yellow and red colours) in the range 0-4000 ps and in the range 4000-10000 ps, as opposed to a higher number of clusters in Cupricyclin-2 (higher RMSD values, yellow and green colours.
